# A Triple Checked Partial Ambiguity Resolution for GPS/BDS RTK Positioning

**DOI:** 10.3390/s19225034

**Published:** 2019-11-18

**Authors:** Liguo Lu, Liye Ma, Wanke Liu, Tangting Wu, Benfu Chen

**Affiliations:** 1 Faculty of Geomatics, East China University of Technology, Nanchang 330013, China; lglu@ecit.cn (L.L.); bfchen@ecit.cn (B.C.); 2 Xi’an Research Institute of Surveying and Mapping, Xi’an 710054, China; 3 State Key Laboratory of Geo-information Engineering, Xi’an 710054, China; 4 GNSS Research Center, Wuhan University, Wuhan 430079, China; 2012301610164@whu.edu.cn; 5 School of Geodesy and Geomatics, Wuhan University, Wuhan 430079, China; wkliu@sgg.whu.edu.cn

**Keywords:** partial ambiguity resolution, bootstrapping success rate, bounded fixed-failure ratio test, baseline precision defect, GPS/BDS RTK positioning

## Abstract

Reliable and accurate carrier phase ambiguity resolution is the key to high-precision Global Navigation Satellite System (GNSS) positioning and application. With the fast development of modern GNSS, the increased number of satellites and ambiguities makes it hard to fix all ambiguities completely and correctly. The partial ambiguity fixing technique, which selects a suitable subset of high-dimensional ambiguities to fix, is beneficial for improving the fixed success rate and reliability of ambiguity resolution. In this contribution, the bootstrapping success rate, bounded fixed-failure ratio test, and the new defined baseline precision defect are used for the selection of the ambiguity subset. Then a model and data dual-driven partial ambiguity resolution method is proposed with the above three checks imposed on it, which is named the Triple Checked Partial Ambiguity Resolution (TC-PAR). The comprehensive performance of TC-PAR compared to the full-fixed LAMBDA method is also analyzed based on several criteria including the fixed rate, the fixed success rate and correct fixed rate of ambiguity as well as the precision defect and RMS of the baseline solution. The results show that TC-PAR could significantly improve the fixed success rate of ambiguity, and it has a comparable baseline precision to the LAMBDA method, both of which are at centimeter level after ambiguities are fixed.

## 1. Introduction

With the gradual updating and construction of the four global navigation satellite systems, the number of satellites in orbit will reach more than 100 in the future, and the frequency of navigation signals will increase to three or even more, providing users with more observation information, which will greatly improve the precision, reliability, and availability of satellite navigation and positioning services [1]. Integer ambiguity resolution is the key problem in achieving high-precision positioning of the GNSS. With an increased number of satellite observation equations, the float ambiguities will have higher precision and be easier to fix accurately. However, this will inevitably lead to an increase in the ambiguity resolution dimension at the same time, which will increase the risk of fixing all ambiguities, thus possibly reducing the fixed success rate. Therefore, the Partial Ambiguity Resolution (PAR), which only fixes a suitable subset of the high-dimensional ambiguity set, may be a better choice.

The idea of PAR was first proposed by Teunissen, and the subset was selected based on the Bootstrapping Success Rate (BSR) criterion, so it is named the Success Rate Criterion (SRC) [2]. As SRC performs well in guaranteeing the fixed success rate of ambiguities, it has been widely used in ambiguity resolution [3,4,5,6,7]. Many other strategies have been proposed to select ambiguity subsets, which can generally be divided into three levels and two categories [8], that is, the satellite level, frequency level, and ambiguity level and the model-driven category and data-driven category. The methods used for the satellite level are mostly based on experience, such as the elevation order strategy [9,10,11,12], the Signal-to-Noise Ratio (SNR) order strategy [13], the continuous tracking epochs strategy [14,15], etc. These methods are easy to realize but may not work very well. The frequency level is mainly determined by the Wide-Lane/Narrow-Lane (WL/NL) cascading strategy [1,16,17,18], which first fixes the WL ambiguities that have relatively higher precision, and then the NL ambiguities are updated, and if possible, are also fixed sequentially. The methods used to determine the ambiguity level usually involve the selection of subsets according to the precision order of each ambiguity, such as SRC and the minimum Ambiguity Dilution Of Precision (ADOP) strategy [13,19]. The model-driven and data-driven categories are distinguished by whether actual observations are used or not [8,20,21,22,23]. A representative data-driven method is the general integer aperture [21,22,23].

Although many studies have been carried out, some problems in PAR have still not been solved well. First, as there are several methods to select an ambiguity subset, most of them are independent from each other and have their own advantages and disadvantages, so it is difficult for users to choose which method to use. In addition, since the purpose of fixing ambiguity is to improve the precision of the baseline vector, its influence on the baseline solution needs to be considered when selecting an ambiguity subset [6,24,25,26]. Therefore, a comprehensive method that includes most of the advantages of the current methods as well as considers the precision improvement at baseline is needed.

In this study, a new partial ambiguity resolution method with multiple checks of both the model-driven and data-driven categories is proposed, and the practical effects are evaluated compared to the full-fixed Least-squares AMBiguity Decorrelation Adjustment (LAMBDA) method. The outline of this paper is as follows: Section 2 describes the basic theories of ambiguity resolution. Section 3 describes the main theories of the proposed method. Section 4 shows the experimental results, and finally, Section 5 draws the main conclusions.

## 2. Theory of Ambiguity Resolution

The GNSS linear observation equation is generally expressed as

(1) $$E\left(y\right)=Aa+Bb+\Delta ,\;D(y)=P_{yy}^{-1}$$

where y is the vector of the carrier phase and code observation, and $P_{yy}$ is the weight matrix. E(y) and D(y) are, respectively, the mathematical expectation and variance of y; a is the vector of integer ambiguities; b is the vector of real-valued parameters such as baseline components; A and B are the corresponding design matrices; and Δ is the vector of measurement noise which is assumed to have a zero-mean Gaussian normal distribution.

Due to the integer nature of ambiguity, Equation (1) is a mixed real-integer model, and it is usually solved by a four-step procedure, including (1) estimation of the float ambiguities and baseline parameters, (2) fixing of the float ambiguities to integer values, (3) validation of the integer ambiguities, and (4) update of the baseline with fixed ambiguities [25].

In step (1), we discard the integer nature of ambiguities and perform a standard least-squares adjustment. As a result, we obtain the real-valued estimates of a and b as well as their variance-covariance matrix:

(2) $$\left(\begin{array}{c} \hat{a}\\ \hat{b} \end{array}\right),\;\left(\begin{array}{cc} Q_{\hat{a}\hat{a}} & Q_{\hat{a}\hat{b}}\\ Q_{\hat{b}\hat{a}} & Q_{\hat{b}\hat{b}} \end{array}\right).$$

In step (2), the integer constraints are taken into account, and the real-valued float ambiguities are fixed to integers. The most extensively used integer estimation methods are Integer Rounding (IR), Integer Bootstrapping (IB) [27] and Integer Least Square (ILS) [28], among which ILS is the optimal method, as it can maximize the probability of correct integer estimation. In this paper, we use ILS estimation, and the following contents are based on its representative method LAMBDA [29,30], which first reduces the correlation of ambiguities by Z-transformation:

(3) $$\hat{z}=Z^{T}\hat{a},\;Q_{\hat{z}\hat{z}}=Z^{T}Q_{\hat{a}\hat{a}}Z$$

where Z is a integer transformation matrix that satisfies two conditions: First, each element of Z should be an integer; second, the determinant value of Z should equal to one. After reduction, the float ambiguities $\hat{z}$ are fixed to integers $\overset{⌣}{z}$ by a fast search procedure, and then an inverse Z-transformation is performed to recover the original ambiguities $\overset{⌣}{a}$.

In step (3), the fixed ambiguities $\overset{⌣}{a}$ are validated with ambiguity accepting tests. If $\overset{⌣}{a}$ is rejected by the acceptance test, it is unreliable and may be an incorrect integer solution, then only a float solution can be used.

If $\overset{⌣}{a}$ is accepted by ambiguity accepting tests, then in step (4), we update the float baseline solution by fixed ambiguities:

(4) $$\;\overset{⌣}{b}(\overset{⌣}{a})=\hat{b}-Q_{\hat{b}\hat{a}}Q_{\hat{a}\hat{a}}^{-1}(\hat{a}-\overset{⌣}{a}).$$

According to the error propagation law, the variance-covariance matrix of $\overset{⌣}{b}(\overset{⌣}{a})$ can be evaluated as follows, which shows the baseline precision promotion when ambiguities are fixed to integers:

(5) $$\;Q_{\overset{⌣}{b}(\overset{⌣}{a})\overset{⌣}{b}(\overset{⌣}{a})}=Q_{\hat{b}\hat{b}}-Q_{\hat{b}\hat{a}}Q_{\hat{a}\hat{a}}^{-1}Q_{\hat{a}\hat{b}}.$$

As for partial ambiguity resolution, we can fix a subset of the full ambiguities set. Suppose that the dimensions of the full ambiguities set and partial ambiguities subset are, respectively, n and p, Equation (3) can be rewritten as

(6) $$\;Z=\left[\begin{array}{cc} Z_{n-p} & Z_{p} \end{array}\right],\;\hat{z}=Z^{T}\hat{a}=\left[\begin{array}{c} {\hat{z}}_{n-p}\\ {\hat{z}}_{p} \end{array}\right].$$

According to Equations (4) and (5), we now get the updated baseline solution $\overset{⌣}{b}({\overset{⌣}{z}}_{p})$ and its variance-covariance matrix $Q_{{\overset{⌣}{b}}_{p}{\overset{⌣}{b}}_{p}}$ with the partial fixed ambiguities subset:

(7) $$\;\overset{⌣}{b}({\overset{⌣}{z}}_{p})=\hat{b}-Q_{\hat{b}{\hat{z}}_{p}}Q_{{\hat{z}}_{p}{\hat{z}}_{p}}^{-1}({\hat{z}}_{p}-{\overset{⌣}{z}}_{p})$$

(8) $$\;Q_{{\overset{⌣}{b}}_{p}{\overset{⌣}{b}}_{p}}=Q_{{\hat{b}}_{p}{\hat{b}}_{p}}-Q_{\hat{b}{\hat{z}}_{p}}Q_{{\hat{z}}_{p}{\hat{z}}_{p}}^{-1}Q_{{\hat{z}}_{p}\hat{b}}$$

where $Q_{\hat{b}{\hat{z}}_{p}}=Q_{\hat{b}\hat{a}}Z_{p}$, $Q_{{\hat{z}}_{p}{\hat{z}}_{p}}=Z_{p}^{T}Q_{\hat{a}\hat{a}}Z_{p}$.

Equation (8) shows the baseline precision promotion when partial ambiguities are fixed to integers [26]. Note that $Q_{{\hat{z}}_{p}{\hat{z}}_{p}}^{}$ is always a positive definite matrix. The second term on the right of Equation (8) will increase with the rise of the ambiguity subset dimension, and $Q_{{\overset{⌣}{b}}_{p}{\overset{⌣}{b}}_{p}}$ will decrease correspondingly, which leads to higher baseline precision. This is significant for the selection of ambiguity subsets, as the baseline precision promotion is directly related to the dimensions of the ambiguity subset. So even in a partial ambiguity resolution, the dimensions of the subset should be as large as possible to get a high enough precision.

### 2.1. A Basic Proof of Equivalence

Similarly, the remaining float ambiguities ${\hat{z}}_{n-p}$ can be updated with the fixed ambiguity subset ${\overset{⌣}{z}}_{p}$:

(9) $$\;{{\hat{z}}^{\prime }}_{n-p}={\hat{z}}_{n-p}-Q_{{\hat{z}}_{n-p}{\hat{z}}_{p}}Q_{{\hat{z}}_{p}{\hat{z}}_{p}}^{-1}({\hat{z}}_{p}-{\overset{⌣}{z}}_{p})$$

(10) $$\;{Q^{\prime }}_{{\hat{z}}_{n-p}{\hat{z}}_{n-p}}=Q_{{\hat{z}}_{n-p}{\hat{z}}_{n-p}}-Q_{{\hat{z}}_{n-p}{\hat{z}}_{p}}Q_{{\hat{z}}_{p}{\hat{z}}_{p}}^{-1}Q_{{\hat{z}}_{p}{\hat{z}}_{n-p}}$$

where ${{\hat{z}}^{\prime }}_{n-p}$ are the conditionally updated float ambiguities, which generally have higher precision than ${\hat{z}}_{n-p}$.

However, there are two questions about whether to consider using ${{\hat{z}}^{\prime }}_{n-p}$ to update the baseline solution [31]. The first one is whether using both ${\overset{⌣}{z}}_{p}$ and ${{\hat{z}}^{\prime }}_{n-p}$ could further improve baseline precision. The second is whether using both ${\overset{⌣}{z}}_{p}$ and ${{\overset{⌣}{z}}^{\prime }}_{n-p}$ (fixing${{\hat{z}}^{\prime }}_{n-p}$ to integers${{\overset{⌣}{z}}^{\prime }}_{n-p}$ by IR estimation) to update baseline solution is effective. Next, we analyze these questions from three aspects.

Case 1: If subset ${\overset{⌣}{z}}_{p}$ is correctly fixed, but the remaining subset ${{\overset{⌣}{z}}^{\prime }}_{n-p}$ is incorrectly fixed, using ${\overset{⌣}{z}}_{p}$ and ${{\overset{⌣}{z}}^{\prime }}_{n-p}$ together to update the baseline solution would cause a large deviation due to existing incorrect ambiguities. The result may be even worse than the real-valued baseline solution using float ambiguity.

Case 2: If the ambiguities in subset ${\overset{⌣}{z}}_{p}$ and the remaining ${{\overset{⌣}{z}}^{\prime }}_{n-p}$ are all correctly fixed, according to Equation (10), using ${\overset{⌣}{z}}_{p}$ and ${{\overset{⌣}{z}}^{\prime }}_{n-p}$ together to update the baseline solution will obtain the largest precision promotion. However, considering the multi-frequency and multi-system background, the ambiguity subsets usually have high dimensions, and a pretty high baseline precision can be obtained when it is updated by the ambiguity subsets. So, it is unnecessary to fix the remaining ambiguities, which may bring about wrong ambiguity fixing and do harm to the baseline precision.

Case 3: Not fixing conditionally updated float ambiguities ${{\hat{z}}^{\prime }}_{n-p}$, but using ${\overset{⌣}{z}}_{p}$ and ${{\hat{z}}^{\prime }}_{n-p}$ together to update the baseline solution is in fact equivalent to directly using ${\overset{⌣}{z}}_{p}$. This can be demonstrated as follows:

For convenience, we set $\hat{z}={\left[\begin{array}{cc} {\hat{z}}_{1} & {\hat{z}}_{2} \end{array}\right]}^{T}$. If we only use the fixing subset ${\overset{⌣}{z}}_{2}$ to update the baseline solution, we have

(11) $$\;\overset{⌣}{b}({\overset{⌣}{z}}_{2})=\hat{b}-Q_{\hat{b}{\hat{z}}_{2}}Q_{{\hat{z}}_{2}{\hat{z}}_{2}}^{-1}({\hat{z}}_{2}-{\overset{⌣}{z}}_{2}).$$

If we use subset ${\overset{⌣}{z}}_{2}$ and the conditionally updated float ambiguity ${{\hat{z}}^{\prime }}_{1}$ to update the baseline solution, we have

(12) $$\;{\overset{⌣}{b}}^{\prime }=\hat{b}-\left[\begin{array}{cc} Q_{\hat{b}{\hat{z}}_{1}} & Q_{\hat{b}{\hat{z}}_{2}} \end{array}\right]{\left[\begin{array}{cc} Q_{{\hat{z}}_{1}{\hat{z}}_{1}} & Q_{{\hat{z}}_{1}{\hat{z}}_{2}}\\ Q_{{\hat{z}}_{2}{\hat{z}}_{1}} & Q_{{\hat{z}}_{2}{\hat{z}}_{2}} \end{array}\right]}^{-1}\left[\begin{array}{c} {\hat{z}}_{1}-{{\hat{z}}^{\prime }}_{1}\\ {\hat{z}}_{2}-{\overset{⌣}{z}}_{2} \end{array}\right]$$

where $\;{{\hat{z}}^{\prime }}_{1}={\hat{z}}_{1}-Q_{{\hat{z}}_{1}{\hat{z}}_{2}}Q_{{\hat{z}}_{2}{\hat{z}}_{2}}^{-1}({\hat{z}}_{2}-{\overset{⌣}{z}}_{2})$.

The second term on the right of the Equation (12) can be reduced as

(13) $$\begin{array}{l} \left[\begin{array}{cc} Q_{\hat{b}{\hat{z}}_{1}} & Q_{\hat{b}{\hat{z}}_{2}} \end{array}\right]{\left[\begin{array}{cc} Q_{{\hat{z}}_{1}{\hat{z}}_{1}} & Q_{{\hat{z}}_{1}{\hat{z}}_{2}}\\ Q_{{\hat{z}}_{2}{\hat{z}}_{1}} & Q_{{\hat{z}}_{2}{\hat{z}}_{2}} \end{array}\right]}^{-1}\left[\begin{array}{c} {\hat{z}}_{1}-{{\hat{z}}^{\prime }}_{1}\\ {\hat{z}}_{2}-{\overset{⌣}{z}}_{2} \end{array}\right]\\ =\left[\begin{array}{cc} Q_{\hat{b}{\hat{z}}_{1}} & Q_{\hat{b}{\hat{z}}_{2}} \end{array}\right]\left[\begin{array}{cc} \;{\tilde{Q}}_{{\hat{z}}_{1}{\hat{z}}_{1}}^{-1} & -{\tilde{Q}}_{{\hat{z}}_{1}{\hat{z}}_{1}}^{-1}Q_{{\hat{z}}_{1}{\hat{z}}_{2}}Q_{{\hat{z}}_{2}{\hat{z}}_{2}}^{-1}\\ -Q_{{\hat{z}}_{2}{\hat{z}}_{2}}^{-1}Q_{{\hat{z}}_{2}{\hat{z}}_{1}}{\tilde{Q}}_{{\hat{z}}_{1}{\hat{z}}_{1}}^{-1} & Q_{{\hat{z}}_{2}{\hat{z}}_{2}}^{-1}+Q_{{\hat{z}}_{2}{\hat{z}}_{2}}^{-1}Q_{{\hat{z}}_{2}{\hat{z}}_{1}}{\tilde{Q}}_{{\hat{z}}_{1}{\hat{z}}_{1}}^{-1}Q_{{\hat{z}}_{1}{\hat{z}}_{2}}Q_{{\hat{z}}_{2}{\hat{z}}_{2}}^{-1} \end{array}\right]\left[\begin{array}{c} Q_{{\hat{z}}_{1}{\hat{z}}_{2}}Q_{{\hat{z}}_{2}{\hat{z}}_{2}}^{-1}({\hat{z}}_{2}-{\overset{⌣}{z}}_{2})\\ {\hat{z}}_{2}-{\overset{⌣}{z}}_{2} \end{array}\right]\\ =\left[\begin{array}{cc} Q_{\hat{b}{\hat{z}}_{1}} & Q_{\hat{b}{\hat{z}}_{2}} \end{array}\right]\left[\begin{array}{c} 0\\ Q_{{\hat{z}}_{2}{\hat{z}}_{2}}^{-1} \end{array}\right]\left({\hat{z}}_{2}-{\overset{⌣}{z}}_{2}\right)\\ =Q_{\hat{b}{\hat{z}}_{2}}Q_{{\hat{z}}_{2}{\hat{z}}_{2}}^{-1}\left({\hat{z}}_{2}-{\overset{⌣}{z}}_{2}\right) \end{array}$$

where $\;{\tilde{Q}}_{{\hat{z}}_{1}{\hat{z}}_{1}}=Q_{{\hat{z}}_{1}{\hat{z}}_{1}}-Q_{{\hat{z}}_{1}{\hat{z}}_{2}}Q_{{\hat{z}}_{2}{\hat{z}}_{2}}^{-1}Q_{{\hat{z}}_{2}{\hat{z}}_{1}}$.

According to Equations (12) and (13), we get ${\overset{⌣}{b}}^{\prime }=\hat{b}-Q_{\hat{b}{\hat{z}}_{2}}Q_{{\hat{z}}_{2}{\hat{z}}_{2}}^{-1}\left({\hat{z}}_{2}-{\overset{⌣}{z}}_{2}\right)=\overset{⌣}{b}({\overset{⌣}{z}}_{2})$, and the equivalence is proved. Thus, in this paper, it is considered that directly updating the baseline using Equations (7) and (8) is a better way of performing partial ambiguity resolution, as this strategy is more reliable, more effective, and simpler than the above three cases, especially in multi-frequency and multi-system backgrounds where the dimension of the ambiguities is generally higher.

Up until now, we have identified the usage of the ambiguity subset. The next question is how to select a proper subset, and this is the key problem in partial ambiguity resolution. Ambiguity subset selection contains two main questions: First, we need to define the screening order of every ambiguity based on certain criteria. Many methods have been used, such as the elevation ordering strategy [9,10,11,12], the ADOP minimization strategy [13,19], and the SRC strategy [2,3,4,5,6,7]. Second, after the order is determined, the size of ambiguity subset needs to be determined as well. This also requires a criterion, for example, the ambiguity accepting test. Aiming at addressing the above two questions, many studies have been carried out, and they can be divided into two categories including the model-driven category and the data-driven category.

### 2.2. Model Driven Ambiguity Subset Selection

If the ambiguity subsets are chosen without taking any measurement, namely only making use of the variance-covariance matrix $Q_{\hat{a}\hat{a}}$ or other empirical information like satellite elevation, then the method used belongs to the model-driven category. Another property of the model-driven category is that the strength of the observation model is usually used to judge whether the fixed ambiguities are correct or not. A representative method is the SRC, which uses the ambiguity fixed success rate as a criterion, and the screening order of SRC is based on the conditional variance of ambiguity.

The essence of choosing ambiguity subsets is screening out the low-precision ambiguities and preserving those with high precision, but we must determine how to judge the precision of each ambiguity. Some empirical methods such as satellite elevation order were used at first, but they lack a certain theoretical basis and may be ineffective in some cases, for example, GEO satellites always have a high elevation but usually have a worse observation quality due to the larger distance. It will also be hard to achieve a good performance using the elevation order if some gross errors exist in it. On the other hand, some linear combination ambiguities with higher relative precision are fixed first, such as the extra wide-lane and wide-lane ambiguity, which is, in fact, a special case of the LAMBDA method. Linear combination is an example of integer bootstrapping [32]. So, the LAMBDA algorithm based on Integer Least Squares Estimation (ILSE) is a better choice for studying partial ambiguity resolution, and a representative method to select the ambiguity subset is applying the order of conditional variance matrix *D*.

A Cholesky decomposition of $\;Q_{\hat{z}\hat{z}}$ can be presented as

(14) $$\;Q_{\hat{z}\hat{z}}=L^{T}DL$$

where *L* is a unit upper triangular, and *D* is a diagonal matrix named the conditional variance matrix.

According to the LAMBDA algorithm, matrix *D* is sorted in descending order as much as possible. The ambiguity searching order goes from the last conditional variance to the first conditional variance. Therefore, when screening an ambiguity subset, we can also adopt this order and select the higher-precision ambiguities at first.

The ambiguity fixed success rate is widely used to determine the reliability of the ambiguity subset, as it reflects the strength of the GNSS observation model to a certain extent as well as quantifies it by a mathematical expression. When the fixed success rate is high enough and, corresponding, the failure rate is low enough, it is considered reliable to accept the ambiguity fixed solution. The SRC is proposed based on this principle.

The ILSE has been proven to have the largest success rate and is considered theoretically optimal [28], but it is difficult to directly perform numerical calculations. However, the BSR is easily calculated and is usually used as the lower boundary of the ILSE [27,33]:

(15) $$\;P_{s\_IB}=\prod_{i=1}^{n}\left(2\Phi (\frac{1}{2\sqrt{d_{i}}})-1\right)$$

where *n* is the dimension of the ambiguities; Φ(·) is the cumulative distribution function of the standard Gaussian normal distribution; and $d_{i}$ represents the diagonal elements of the conditional variance matrix *D*. Note that *D* is decomposed from the variance-covariance matrix $Q_{\hat{z}\hat{z}}$ after decorrelation.

SRC selects ambiguity subsets by setting a certain threshold of ε (e.g., ε=0.995). First, it calculates the $P_{s\_IB}$ of the full ambiguity set and compares it with the given threshold ε. If it is smaller than ε, then sequentially remove the ambiguity with a small subscript and recalculates the $P_{s\_IB}$ of the remaining ambiguities. This process is repeated until the BSR of the ambiguity subset is larger than or equal to ε, or we end up with a minimal admissible subset (usually the subset size is set to four). The discriminant condition based on SRC can be expressed as follows:

(16) $$If\;\left\{\begin{array}{c} P_{s\_IB}<\varepsilon \;use\;\hat{z}\\ P_{s\_IB}\geq \varepsilon \;use\;\overset{⌣}{z} \end{array}\right..$$

According to the process mentioned above, we may find SRC to be a simple and easy-to-use method, as $P_{s\_IB}$ can be calculated quickly as soon as we obtain the decorrelated matrix $Q_{\hat{z}\hat{z}}$, and the decision as to whether or not to accept the fixed ambiguities can be made prior to the integer estimation step, which is very timesaving. Besides, it is also benefit for users to have a global knowledge of the GNSS observation model as well as a quality description of the ambiguities and baseline solution [34].

**Figure 1:**
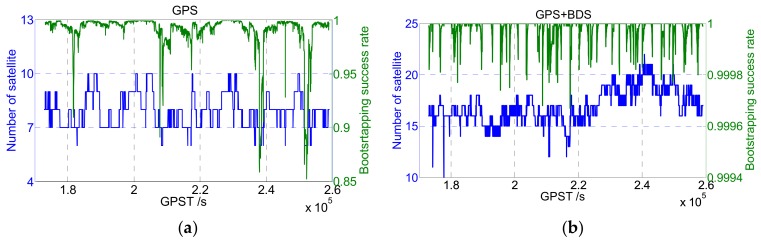
Bootstrapping success rate of GPS (**a**) and GPS+BDS (**b**) dual-frequency data.

However, in multiple frequencies and multiple system scenes where more observations are available, the increase of satellite number will improve the strength of the observation model, as well as the value of BSR. An experiment was applied to illustrate the change of BSR value: The BSR values of GPS and GPS+BDS were calculated based on real-measured dual-frequency data (cf. Figure 1), respectively. As shown in Figure 1, the BSR of some epochs is low when only the GPS system is adopted, and SRC may achieve good performance by screening out individual ambiguities with low precision. However, when GPS + BDS is adopted, the BSR is always close to 100% due to the enhancement of the model’s strength. Thus, in the multiple frequencies and multiple systems scene, the BSR indicator may not continue to be used effectively for ambiguity screening, and other strategies like data-driven methods need to be performed together.

### 2.3. Data Driven Ambiguity Subset Selection

Different from the model-driven category, if the ambiguity subsets are chosen with actual measurements involved, then the method belongs to the data-driven category. Almost all the ambiguity accepting tests belong to this category, such as the ratio test [35,36,37], the difference test [38,39], and the project test [40].

Once the ambiguity subset ${\hat{z}}_{p}$ is fixed to integer vector ${\overset{⌣}{z}}_{p}$, an acceptance test is performed to validate the reliability of this subset. Taking the most widely used ratio test as an example, suppose the threshold of the ratio test is expressed by ‘c’ (it could be 2.5, 3, or other suitable values), then the discriminant condition can be expressed as follows:

(17) $$If\;\frac{\left({\hat{z}}_{p}-{\overset{⌣}{z}}_{p,2})Q_{{\hat{z}}_{p}{\hat{z}}_{p}}^{-1}({\hat{z}}_{p}-{\overset{⌣}{z}}_{p,2}\right)}{\left({\hat{z}}_{p}-{\overset{⌣}{z}}_{p})Q_{{\hat{z}}_{p}{\hat{z}}_{p}}^{-1}({\hat{z}}_{p}-{\overset{⌣}{z}}_{p}\right)}\;\left\{\begin{array}{c} \geq c\;use\;{\overset{⌣}{z}}_{p}\\ <c\;use\;{\hat{z}}_{p} \end{array}\right.$$

where ${\overset{⌣}{z}}_{p,2}$ is the suboptimal candidate.

As Equation (17) shows, the ratio test is constructed by the second minimum and minimum quadratic form of the ambiguity residual, which is obviously a data-driven method, as the ambiguities calculated from actual observations are considered. The subset ${\overset{⌣}{z}}_{p}$ will be used to update the baseline solution if it is accepted by the ratio test, or furthermore, ambiguities need to be screened out if ${\overset{⌣}{z}}_{p}$ is rejected.

The key problem of the ratio test is the selection of the threshold c, which is normally an empirical value such as 2.5 or 3 [41,42]. However, this empirical value will no longer be applicable in multi-frequency and multi-system cases, as the difference between the second minimum and minimum quadratic form will no longer be significant with the increase in the ambiguity dimension, and the ratio value will gradually approach to one. As a result, the empirical threshold of 2.5 or 3 may be too strict in high-dimension cases, and subset ${\overset{⌣}{z}}_{p}$ will be continuously rejected until the size of the subset becomes very low, which is not conducive to the improvement of baseline precision.

FFRT provides a more reasonable method to determine threshold c. An adaptable threshold is calculated according to the underlying model (measured by BSR) and failure rate tolerance. We calculate the ratio value of 5 to 65 dimensions using the Monte Carlo method with 100,000 samples, and the BSR is set from 0.5 to 1. The failure rate tolerance is set to 0.001. Then, a look-up table is established for convenience, which is like the table published by Verhagen [37].

**Figure 2:**
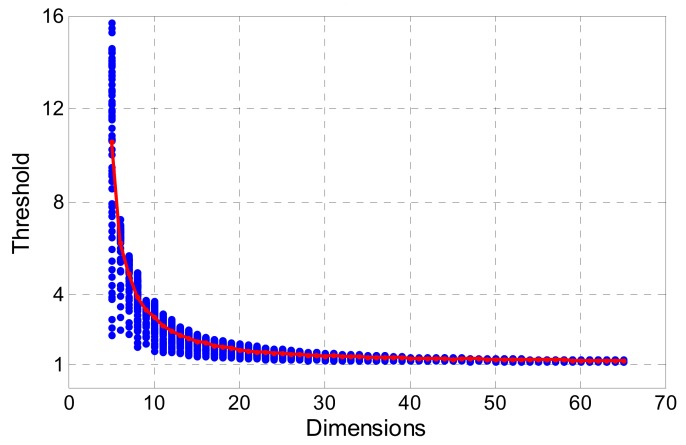
A plot of the threshold under FFRT as function of ambiguity dimension. Blue denotes thresholds calculated at different BSR values and dimensions; red denotes the mean threshold of each dimension.

As Figure 2 shows, the testing threshold calculated by FFRT is more sensitive to the dimension of ambiguity, as it is larger in low-dimension cases and smaller in high-dimension cases. An empirical value like 2.5 or 3 is more suitable for single-system cases where the dimension is around 10, and it is not suitable for the multi-frequency and multi-system cases. In this paper, we adopt the FFRT method for screening ambiguity subsets, and an improvement is made on it.

**Figure 3:**
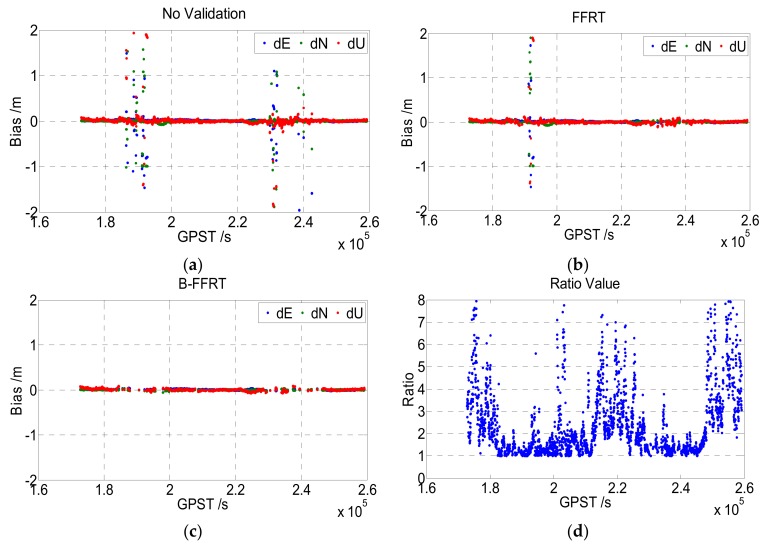
Baseline bias obtained by No-Validation (**a**), FFRT (**b**), B-FFRT (**c**) respectively, and the ratio value (**d**) of it.

Note that the thresholds calculated by FFRT are not strictly accurate, as they are influenced by the BSR and simulated results, which may be overoptimistic compared with the real case, so the threshold is close to one when ambiguity has a high dimension or the underlying model is very strong. As a missed detection may occur if the testing threshold is too small, in order to guarantee the reliability of fixed ambiguity subsets, the testing threshold is set to a constant value of 1.5 when it is smaller than 1.5. This modified method is named the Bounded-FFRT (B-FFRT). An experiment was applied to compare the FFRT and B-FFRT. Real-measured GPS+BDS dual-frequency data were processed by LAMBDA and then validated by FFRT and B-FFRT respectively, and an ambiguity fixing with no validation (the threshold of ratio is set as 1) is also evaluated as a comparison (cf. Figure 3).

As Figure 3 shows, the FFRT method really makes it more reasonable compared to an empirical threshold, but some wrongly fixed ambiguities still exist compared to B-FFRT. Combined with the ratio value, we can see that wrong fixing is more likely to arise when the ratio is small. As the threshold of FFRT is calculated by simulation experiments, it may not be completely in line with the actual situation, so a missed detection may happen when the calculated threshold is too small. We evaluated the missed detection rate and false alarm rate (cf. Table 1); the missed detection rate of No-Validation, FFRT, and B-FFRT is 11.74%, 6.88%, and 0.69%, respectively, and the false alarm rate is 0%, 5.63%, 31.11%, respectively. Although the false alarm rate of B-FFRT is much larger than FFRT, the missed detection rate is also much smaller, which may realize higher reliability and perform better in an actual situation.

## 3. A New Model-Driven and Data-Driven Partial Ambiguity Resolution Method

### 3.1. The Baseline Precision Defect (BPD)

The main contribution and purpose of fixing ambiguities is to improve the precision of the baseline solution. For instance, in a single-epoch processing mode, the float solution and fixed solution of the baseline vector can be expressed as

(18) $$\;Q_{\hat{b}\hat{b}}\;=\;\delta_{p}^{2}{\left(G^{T}PG\right)}^{-1}$$

(19) $$\;Q_{\overset{⌣}{b}\overset{⌣}{b}}\;\approx \;\delta_{\varphi }^{2}{\left(G^{T}PG\right)}^{-1}$$

where $\delta_{p}^{}$ and $\delta_{\varphi }^{}$ are the priori accuracy of the code and carrier phase observations, respectively. G is the direction of the cosine matrix of satellites and receiver line-sight. The detailed proof of Equations (18) and (19) is given in Appendix A.

According to Equations (18) and (19), the precision of the float baseline solution mainly depends on the code observations, while the fixed baseline solution depends on the carrier phase observations, so the precision of the baseline can obtain great promotion after the ambiguities have been fixed. Furthermore, as presented in Equation (10), the larger the dimension of the ambiguity subset is, the higher the baseline precision is, so when choosing an ambiguity subset, its influence on baseline precision should also be considered. In order to fully evaluate the baseline precision after fixing the partial ambiguities, the Baseline Precision Defect (BPD) is defined in this paper as follows, and a similar concept can also be found in Teunissen [24] and Hou [6]:

(20) $$\;BPD=\sqrt{\frac{tr(Q_{\hat{b}\hat{b}})}{tr(Q_{\overset{⌣}{b}\overset{⌣}{b}})}}-\sqrt{\frac{tr(Q_{\hat{b}\hat{b}})}{tr(Q_{\overset{⌣}{b}({\overset{⌣}{z}}_{p})\overset{⌣}{b}({\overset{⌣}{z}}_{p})})}}$$

where $Q_{\hat{b}\hat{b}}$, $Q_{\overset{⌣}{b}\overset{⌣}{b}}$, and $Q_{\overset{⌣}{b}({\overset{⌣}{z}}_{p})\overset{⌣}{b}({\overset{⌣}{z}}_{p})}$ represent the variance-covariance matrix of the float baseline, fixed baseline (updated by the full ambiguities set), and partial fixed baseline (updated by the ambiguity subset), respectively. tr(·) is the matrix tracing operation.

The first term on the right of Equation (20) reflects the baseline precision promotion of the full ambiguity set, and the second term reflects the promotion of the ambiguity subset, so the subtraction of them reflects the precision defect caused by partial ambiguity fixing, which is better when it is smaller. Suppose that the a priori accuracy of the code and carrier phase observations is 0.3 and 0.003 m, respectively, then the first right term will be approximately equal to 100 and the second right term will be less than (when the dimension of the subset is smaller than that of the full set) or approximately equal to (the subset is equal to the full set) 100. As the baseline solution will obtain a larger precision with a smaller BPD, the influence of partial ambiguity fixing can be quantitatively evaluated easily through BPD. Hence, the upper boundary value of BPD (50 for example) can be set while choosing ambiguity subsets to ensure high enough baseline precision promotion.

**Figure 4:**
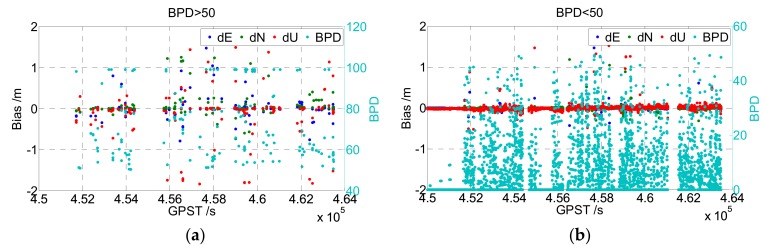
Baseline bias obtained with BPD > 50 (**a**) and BPD < 50 (**b**).

An experiment was applied to illustrate the beneficial effect of the BPD check. Partial ambiguity fixing was performed on real-measured GPS+BDS dual-frequency data, and the result of the baseline vector was compared with its real value (cf. Figure 4). The left figure of Figure 4 shows the results that satisfied BPD < 50. Most of them were wrongly fixed. The right figure shows those that satisfied BPD < 50, in which only little wrong fixing occurred.

### 3.2. The Triple Checked Partial Ambiguity Resolution (TC-PAR) Method

From the above analysis, we conclude that the model-driven method SRC gives an overall description of the GNSS observation model and an ambiguities/baseline solution with high precision, while the modified data-driven method B-FFRT can distinguish among ambiguity candidates. Combining these two methods would better ensure the reliability of ambiguity subsets. In addition, the newly defined data-driven criteria, BPD, could ensure that the baseline precision promotion is high enough after the ambiguity subset is fixed.

**Figure 5:**
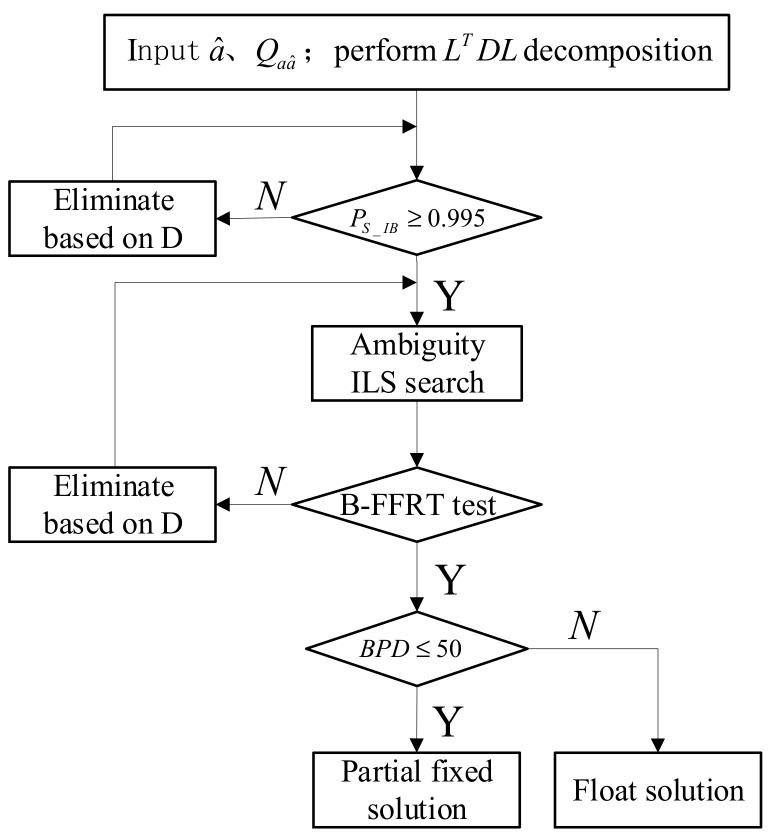
Flow chart of Triple Checked Partial Ambiguity Resolution (TC-PAR).

According to the analysis above, we propose a new partial ambiguity resolution method named Triple Checked Partial Ambiguity Resolution (TC-PAR), which can achieve high reliability as well as a baseline solution with high precision. The flow chart is shown in Figure 5, and the detailed steps are given as follows:

Step 1: Using the order of the conditional variance matrix *D* as a screening order of ambiguity subsets, suppose an $L^{T}DL$ decomposition of $\;Q_{\hat{z}\hat{z}}$ is performed and $d_{1},d_{2},...,d_{n}$ are, respectively, the diagonal elements of *D* ($d_{1}$ is the first one and $d_{n}$ the last one), then the ambiguity corresponding to $d_{1}$ has the lowest relative accuracy. If the full ambiguity set cannot be fixed, it will be screened out from $d_{1}$ until the residual subsets can be fixed one by one.

Step 2: The model-driven SRC method is used as the first check to ensure the strength of the GNSS observation model as well as a high enough fixed success rate of ambiguities. The threshold condition of BSR is set as $P_{s\_IB}\geq 0.995$ in this step, and the ambiguities are eliminated one by one according to the order of Step 1 until this condition is satisfied.

Step 3: After the ambiguity subset is fixed by ILS search, the data-driven B-FFRT method is used as the second check to ensure the reliability of the fixed ambiguity subset. The established B-FFRT look-up table is adopted in this step with the minimum of ratio threshold set as 1.5.

Step 4: The data-driven BPD method is used as the third check to ensure the baseline solution can obtain high enough precision promotion after the ambiguity subset is fixed. The threshold condition of BPD is set as BPD≤50 in this step. The ambiguity subset will be obtained if this condition can be met; otherwise, only a float solution can be used.

## 4. Results

**Figure 6:**
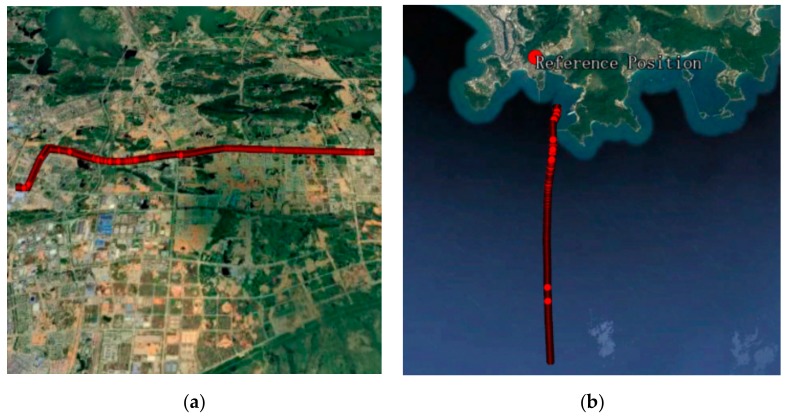
Dynamic trajectory of Dataset3 (**a**) and Dataset4 (**b**).

In order to validate the performance of TC-PAR, four groups of real-measured data in different scenes and different baseline lengths are adopted. These data are processed with two strategies: (a) the traditional LAMBDA method of fixing the full ambiguity with an empirical threshold 2.5; (b) the proposed TC-PAR method of fixing the partial ambiguities subset. The detailed information of these data is given in Table 2, where Dataset1 and Dataset2 are static and Dataset3 and Dataset4 are kinematic. The dynamic trajectory of Dataset3 and Dataset4 is shown in Figure 6.

The basic configuration of data processing is given in Table 3. The dual-frequency and dual-system data of GPS/BDS, which can reach approximately 40 dimensions, are used to satisfy the features of high-dimensional ambiguity resolution. In order to better evaluate the real accuracy of each ambiguity, a single-epoch geometry-based double-difference RTK technique is adopted (Kalman filter is only applied in the estimation of float baseline parameters, and the float ambiguities are calculated by the combination of pseudo-range and carrier phase observations in each epoch). The residual ionosphere and troposphere are corrected by the model, the cut-off angle is set as 10°, and the weight of the code/phase is set as 1:100.

Several criteria are evaluated in this section, including the fixed rate, the fixed success rate, correct fixed rate of ambiguity resolution, as well as the Root Mean Square (RMS) of baseline positions and the BPD value. The fixed rate, fixed success rate and correct fixed rate are defined as follows:

Fixed rate: the ratio of the number of fixed ambiguities to all effective epochs. Note that both correctly fixed and wrongly fixed epochs participate.

Fixed success rate: the ratio of the number of correctly fixed ambiguities to all effective epochs, which reflects the availability of fixed ambiguities. The correctness of ambiguities is evaluated by comparing the ambiguity-fixed positions with the real positions, for instance, the ambiguities are considered correct if the deviations of E/N/U are less than 3 cm/3 cm/6 cm in static scene and 5 cm/5 cm/10 cm in kinematic scene, respectively.

Correct fixed rate: the ratio of the number of correctly fixed ambiguities to all fixed epochs, which reflects the reliability of fixed ambiguities.

### 4.1. Experiment on Static Data

**Figure 7:**
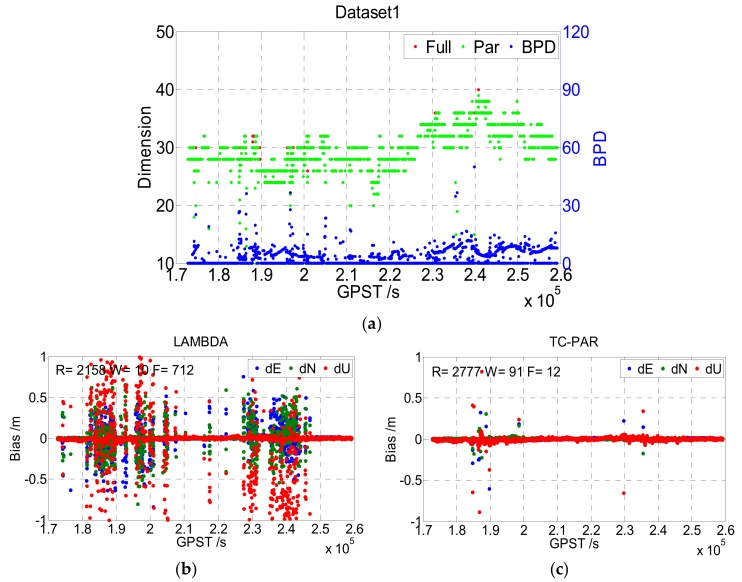
Dimensions of the ambiguity full set/subset and BPD value (**a**), and the baseline deviation sequence of LAMBDA (**b**) and TC-PAR (**c**) in Dataset1. R, W, and F denote the right fixed epoch, wrong fixed epoch, and float epoch, respectively.

**Figure 8:**
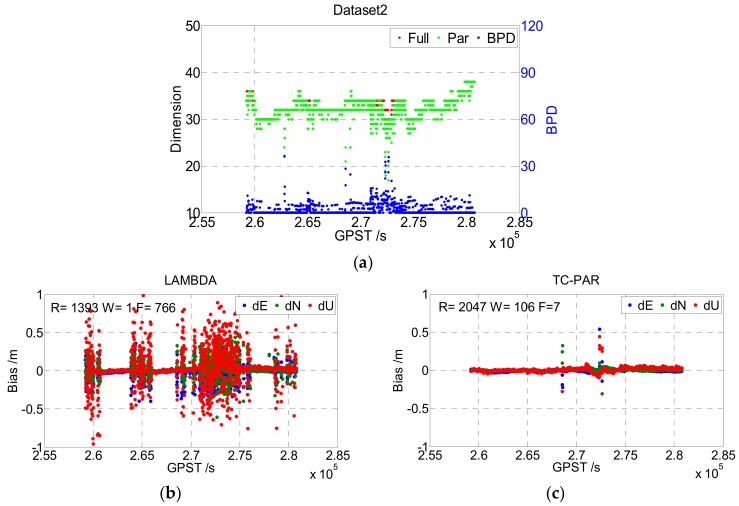
Dimensions of the ambiguity full set/subset and BPD value (**a**), and the baseline deviation sequence of LAMBDA (**b**) and TC-PAR (**c**) in Dataset2. R, W, and F denote the right fixed epoch, wrong fixed epoch, and float epoch, respectively.

In this experiment, the above types of two static data were processed according to Table 3, with the LAMBDA and TC-PAR methods, respectively. The results are shown in Figure 7 and Figure 8. Note that for static data, the real values of baseline positions were calculated by the commercial software CHC Geomatics Office (CGO) developed by CHCNAV, China. As the true ambiguity values were unknown, the ambiguity-fixed baseline positions were compared to determine whether the ambiguities had been fixed correctly.

The top figure of Figure 7 gives the dimensions of the full ambiguity set and partial ambiguity subset with red and green points, respectively, and the BPD of ambiguity subset is also presented with blue points. It can be seen that the dimension of the ambiguity subsets is equal to or slightly lower than that of the full set in most of the epochs, where the first case (equal to) means that the ambiguity full set could be fixed by LAMBDA, and the second case (lower than) means that the unfixed ambiguities could be fixed after screening out a little part. This phenomenon is consistent with the real case, as usually, only a few of the ambiguities/phase observations are abnormal. Additionally, the BPD value, which is better when it is lower, was lower than 20 in most epochs, reflecting a high improvement in baseline precision.

The bottom figures of Figure 7 show the deviation sequence of the baseline vector processed by LAMBDA (b) and TC-PAR (c), respectively, and the epoch numbers of right fixed and wrong fixed are given in the figure. The right fixed epoch of LAMBDA was 2158, and it increased to 2777 when processed by TC-PAR while the float epoch decreased from 712 to 12. Hence, most of the float epochs could be fixed after adopting partial ambiguity fixing; thus, the availability of the fixed solution evidently improved. Figure 8 shows the results of Dataset2; note that the ionosphere of Hong Kong is more active than that of Wuhan, which may explain the worse results of this dataset compared to Dataset1. As the general characteristics of Dataset2 are like Dataset1, no more explanations will be given here.

The statistical results of Dataset1 and Dataset2 are given in Table 4, including the fixed rate, fixed success rate, correct fixed rate, and the RMS of the fixed solution of the baseline vector (including correctly fixed solutions and wrongly fixed solutions) as well as RMS of the fixed and float solution (including both fixed solutions and float solutions). Compared to LAMBDA, TC-PAR was shown to significantly improve the fixed rate and fixed success rate; for instance, the fixed success rates of Dataset1 and Dataset2 improved from 74.93% and 64.49% to 96.42% and 94.77%, respectively. As the float RTK solutions are generally in decimeter accuracy, the baseline RMS of LAMBDA method is in decimeter accuracy when considering float epochs, while TC-PAR could generally maintain centimeter accuracy. The correct fixed rates are decreased to a certain degree, but even though decreased, the correct fixed rates are still higher than 95%, and the RMS of baseline when ambiguities are fixed is comparable to LAMDBA. As partial ambiguity resolution is exactly an attempt at solving the problems of those epochs that are hard to overcome, we think the decrease is unavoidable, and it could be accepted considering the significant improvement of fixed success rate.

### 4.2. Experimental of Kinematic Data

**Figure 9:**
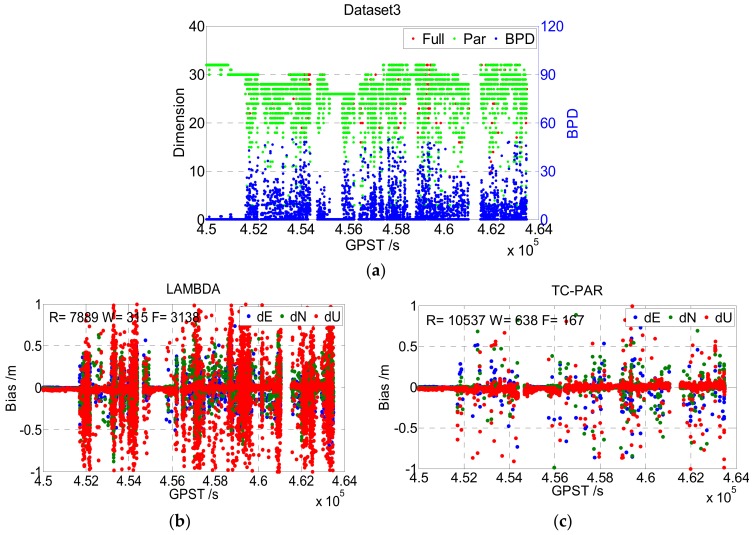
Dimension of ambiguity full set/subset and BPD value (**a**), and the baseline deviation sequence of LAMBDA (**b**) and TC-PAR (**c**) in Dataset3. R, W, and F denote the right fixed epoch, wrong fixed epoch, and float epoch, respectively.

**Figure 10:**
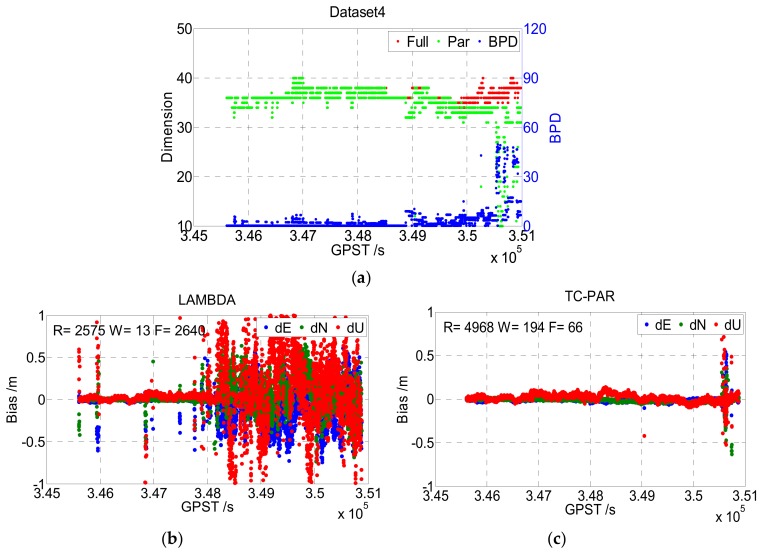
Dimensions of the ambiguity full set/subset and BPD value (**a**) and the baseline deviation sequence of LAMBDA (**b**) and TC-PAR (**c**) in Dataset4. R, W, and F denote the right fixed epoch, wrong fixed epoch, and float epoch, respectively.

In this experiment, the above two types of kinematic data were processed according to Table 3 with the LAMBDA and TC-PAR methods, respectively. The results are shown in Figure 9 and Figure 10. For kinematic data, the real values were calculated by the commercial software GrafMov developed by NovAtel, Calgary, CA. And only the fixed solution of GrafMov (Quality number = 1) was adopted.

Different from the static data, the overall quality of the kinematic data was worse due to the complex environment. It can be seen from Figure 9 that the number of satellites showed large fluctuations, as the car was frequently shaded by nearby buildings and trees. According to the statistical results given in Table 5, the fixed rate of Dataset3 was only 72.33% when processed by LAMDBA, although its baseline length was less than 6 km. However, when processed by TC-PAR, the fixed rate of Dataset3 improved to 98.53%, and the fixed success rate improved from 69.56% to 92.90%, which is as significant as the results shown for the static data. The worse quality of observation of course had an adverse influence on TC-PAR, too. For instance, the BPD value was obviously larger than that in the static case, which means that more ambiguities were screened out due to low accuracy. So, according to practical experience, the discriminant condition of BPD≤50 was set to guarantee the reliability and precision of solutions.

Figure 10 shows the results of Dataset4, which were collected at sea in an open environment. It can be seen that the fixed rate of Dataset4 was very low when processed by LAMBDA, especially after GPST 348000 s. Note that the baseline length of Dataset4 continuously increased, and the reason for this is obvious: The residual error for factors such as the ionosphere and troposphere get larger with the increase of the baseline and is absorbed into the float ambiguities, causing the ratio of Equation (17) to become insignificant. This phenomenon reflects another shortage of the ratio test, as the ratio value itself will be insignificant if the float ambiguities have low accuracy. According to Figure 10 and Table 5, the proposed TC-PAR method, which adopts B-FFRT rather than the traditional ratio test, obtained a higher fixed success rate as well as comparable baseline precision when ambiguities were fixed, and higher baseline precision when float solutions were also considered. However, as shown in the top figure of Figure 10, the dimensions of the ambiguity subsets decreased drastically after GPST 350500, due to the baseline length being too large so that even B-FFRT could not tolerate it anymore.

## 5. Discussion and Conclusions

The experiment results presented in Section 4 show that both the fixed rate and fixed success rate obtained significant improvements when the TC-PAR method was adopted, and although the correct fixed rate is decreased to a certain degree, it is still higher than 95% in the static experiment and higher than 94% in the kinematic experiment. The precision of the baseline solution still maintained a centimeter level after the ambiguity subsets were fixed, which is comparable to the LAMBDA method, and if float solutions are also considered, the precision can be increased from decimeter level to centimeter level. These facts above illustrate the excellent properties of TC-PAR, and the benefits can be classified into three categories:

First is the screening order of ambiguities. The order of the conditional variance matrix *D* can reflect the precision order of each ambiguity effectively. As shown in Figure 7, Figure 8, Figure 9 and Figure 10, the dimensions of ambiguity subsets are slightly lower than that of the full set most of the time, as the low precision ambiguities are identified and eliminated accurately.

Second, the double checks imposed by both model-driven (BSR) and data-driven methods (B-FFRT) guarantee the high reliability of fixed ambiguities. Thus, the fixed rate and fixed success rate of the ambiguities are high.

Third, checked by the new defined data-driven criterion, BPD, the precision of the baseline solution can be maintained at a high level to obtain a precision comparable to that of fixing full ambiguities.

However, although TC-PAR performs well most of the time, some shortages still exist. As we can see from the above experiments, the dimensions of the subsets are low in some epochs and may even reach 4 (the minimum value set for ambiguity resolution) at times. This phenomenon is abnormal in multi-frequency and multi-system cases. We consider that there are two main reasons for this situation: First, as analyzed in the experiment of Dataset4, due to the inherent characteristics of the ratio test, even the B-FFRT method cannot always perform well if the precision of most ambiguities is at a low level, so another acceptance test method with better features is needed. Besides, as the conditional variance matrix *D* is decomposed from the variance-covariance matrix $Q_{\hat{z}\hat{z}}$, which is largely determined by mathematical models, the order of *D* may be inaccurate if the mathematical model is inappropriate. Hence, the anomaly of TC-PAR may be caused by this inaccurate order; thus, the mathematical model needs to be further refined if a better performance is requested.

In conclusion, partial ambiguity resolution is one of the hottest topics in GNSS ambiguity resolution and remains an open problem. Through the theoretical analysis and experiment results above, the new proposed TC-PAR method was shown to achieve a greater fixed success rate with high reliability and high accuracy, which may be beneficial to the future application of GNSS positioning.

## Figures and Tables

**Table 1:** Comparation of missed detection rate and false alarm rate.

Method	Fixed Epochs	Missed Detection Rate	False Alarm Rate
No-Validation	2880	11.74%	0%
FFRT	2578	6.88%	5.63%
B-FFTR	1666	0.69%	31.11%

**Table 2:** Data information.

Dataset	Scene	Date	Instruments	Length
1	Static (WHDH-WHKC)	23 January 2018	Trimble NetR9	18.6 km
2	Static (HKLM-HKQT)	5 July 2017	Trimble NetR9	12.5 km
3	Vehicle	13 March 2015	Sinan M300	0.1~5.7 km
4	Shipborne	7 November 2015	Novatel OME6	5~27 km

**Table 3:** Basic processing configuration.

System and Frequency	GPS(L1/L2)+BDS(B1/B2)
Process Model	Single-epoch RTK
Ambiguity Resolution	LAMBDA/TC-PAR
Validation Threshold	2.5/B-FFRT
Cut-off Angle	10°
Ionosphere Model	Klobuchar
Troposphere Model	Saastamoinen
Weight of Code and Phase	1:100

**Table 4:** Static experiment results of LAMBDA and TC-PAR.

	Method	Fixed Rate	Fixed Success Rate	Correct Fixed Rate	Fixed Epochs			Fixed and Float Epochs		
					RMS-E/cm	RMS-N/cm	RMS-U/cm	RMS-E/cm	RMS-N/cm	RMS-U/cm
Dataset1	LAMBDA	75.28%	74.93%	99.54%	0.75	0.60	1.42	10.83	11.60	36.01
	TC-PAR	99.58%	96.42%	96.83%	0.80	0.83	1.87	1.87	1.31	4.89
Dataset2	LAMBDA	64.54%	64.49%	99.93%	1.27	0.88	2.53	8.04	9.17	19.67
	TC-PAR	99.68%	94.77%	95.08%	1.76	1.28	2.80	1.91	1.71	3.18

**Table 5:** Kinematic experiment results of LAMBDA and TC-PAR.

	Method	Fixed Rate	Fixed Success Rate	Correct Fixed Rate	Fixed Epochs			Fixed and Float Epochs		
					RMS-E/cm	RMS-N/cm	RMS-U/cm	RMS-E/cm	RMS-N/cm	RMS-U/cm
Dataset3	LAMBDA	72.33%	69.56%	96.16%	0.66	0.70	2.22	9.64	11.65	27.37
	TC-PAR	98.53%	92.90%	94.29%	0.73	0.72	2.31	3.77	5.92	16.41
Dataset4	LAMBDA	49.50%	49.25%	99.50%	1.52	1.08	3.82	17.46	16.48	36.73
	TC-PAR	98.74%	95.03%	96.24%	1.76	2.20	3.93	2.95	4.14	7.31

## Appendix A

**Proof of Equations (18) and (19).** According to Equation (1), its normal equation can be presented as

(A1) $$\left[\begin{array}{cc} A^{T}P_{yy}A & A^{T}P_{yy}B\\ B^{T}P_{yy}A & B^{T}P_{yy}B \end{array}\right]\left[\begin{array}{c} \hat{a}\\ \hat{b} \end{array}\right]=\left[\begin{array}{c} A^{T}P_{yy}y\\ B^{T}P_{yy}y \end{array}\right].$$

Then, the variance-covariance matrix of the float ambiguity $\hat{a}$ and baseline $\hat{b}$ can be calculated as

(A2) $$Q={\left[\begin{array}{cc} A^{T}P_{yy}A & A^{T}P_{yy}B\\ B^{T}P_{yy}A & B^{T}P_{yy}B \end{array}\right]}^{-1}=\left[\begin{array}{cc} Q_{\hat{a}\hat{a}} & Q_{\hat{a}\hat{b}}\\ Q_{\hat{b}\hat{a}} & Q_{\hat{b}\hat{b}} \end{array}\right].$$

When code and phase observations are both used, suppose that $y={\left[\begin{array}{cc} l_{f} & l_{p} \end{array}\right]}^{T}$, $A={\left[\begin{array}{cc} \lambda I_{n} & 0 \end{array}\right]}^{T}$, $B={\left[\begin{array}{cc} G^{T} & G^{T} \end{array}\right]}^{T}$, and $P_{yy}=diag(P/\delta_{\phi }^{2},P/\delta_{P}^{2})$. Then, the precision of the float baseline can be presented as

(A3) $$Q_{\hat{b}\hat{b}}\;={\left[B^{T}P_{yy}B-B^{T}P_{yy}A{\left(A^{T}P_{yy}A\right)}^{-1}A^{T}P_{yy}B\right]}^{-1}=\delta_{p}^{2}{\left(G^{T}PG\right)}^{-1}.$$

Similarly, after ambiguities fix the integers, the precision of fixed baseline can be presented as

(A4) $$Q_{\overset{⌣}{b}\overset{⌣}{b}}={\left(B^{T}P_{yy}B\right)}^{-1}=\frac{\delta_{\varphi }^{2}}{1+\delta_{\varphi }^{2}/\delta_{p}^{2}}{\left(G^{T}PG\right)}^{-1}\approx \delta_{\phi }^{2}{\left(G^{T}PG\right)}^{-1}.$$

End of proof. □
